# Functional connectivity in reward circuitry and symptoms of anhedonia as therapeutic targets in depression with high inflammation: evidence from a dopamine challenge study

**DOI:** 10.1038/s41380-022-01715-3

**Published:** 2022-08-04

**Authors:** Mandakh Bekhbat, Zhihao Li, Namrataa D. Mehta, Michael T. Treadway, Michael J. Lucido, Bobbi J. Woolwine, Ebrahim Haroon, Andrew H. Miller, Jennifer C. Felger

**Affiliations:** 1 Department of Psychiatry and Behavioral Sciences, Emory University, Atlanta, GA 30322 USA; 2 School of Psychology and Sociology, Shenzhen University, Shenzhen, 518060 Guangdong Sheng China; 3 Neuroscience Graduate Program, Graduate Division of Biological and Biomedical Sciences, Emory University, Atlanta, GA 30322 USA; 4 Department of Psychology, Emory University, Atlanta, GA 30322 USA; 5 The Winship Cancer Institute, Emory University, Atlanta, GA 30322 USA

**Keywords:** Neuroscience, Depression

## Abstract

Increased inflammation in major depressive disorder (MDD) has been associated with low functional connectivity (FC) in corticostriatal reward circuits and symptoms of anhedonia, relationships which may involve the impact of inflammation on synthesis and release of dopamine. To test this hypothesis while establishing a platform to examine target engagement of potential therapies in patients with increased inflammation, medically stable unmedicated adult MDD outpatients enrolled to have a range of inflammation (as indexed by plasma C-reactive protein [CRP] levels) were studied at two visits involving acute challenge with the dopamine precursor levodopa (L-DOPA; 250 mg) and placebo (double-blind, randomized order ~1-week apart). The primary outcome of resting-state (rs)FC in a classic ventral striatum to ventromedial prefrontal cortex reward circuit was calculated using a targeted, a priori approach. Data available both pre- and post-challenge (*n* = 31/40) established stability of rsFC across visits and determined CRP > 2 mg/L as a cut-point for patients exhibiting positive FC responses (post minus pre) to L-DOPA versus placebo (*p* < 0.01). Higher post-L-DOPA FC in patients with CRP > 2 mg/L was confirmed in all patients (*n* = 40) where rsFC data were available post-challenge (*B* = 0.15, *p* = 0.006), and in those with task-based (tb)FC during reward anticipation (*B* = 0.15, *p* = 0.013). While effort-based motivation outside the scanner positively correlated with rsFC independent of treatment or CRP, change in anhedonia scores negatively correlated with rsFC after L-DOPA only in patients with CRP > 2 mg/L (*r* = -0.56, *p* = 0.012). FC in reward circuitry should be further validated in larger samples as a biomarker of target engagement for potential treatments including dopaminergic agents in MDD patients with increased inflammation.

## Introduction

A significant portion of patients with major depressive disorder (MDD) reliably exhibit evidence of increased inflammation, which has received considerable attention as one pathophysiologic pathway contributing to symptoms of depression and particularly anhedonia [1–5]. Neuroimaging work has revealed that exogenous administration of inflammatory cytokines or cytokine inducers (e.g., endotoxin, vaccination) in a laboratory environment alters activation of and functional connectivity (FC) between reward-related brain regions relevant to reduced motivation and anhedonia [6–11]. Preclinical and clinical evidence suggests that these functional MRI (fMRI) findings may be due to the impact of inflammation on the availability and release of dopamine, in part through limiting its synthetic precursors [12–14]. For example, patients receiving inflammatory cytokine therapy for hepatitis C virus exhibited a pattern of striatal fluorodopa (18F) uptake consistent with reduced dopamine availability and release that correlated with symptoms of depression including reduced motivation [8]. Our work in non-human primates also demonstrated that peripheral inflammatory cytokine-induced decreases in striatal dopamine release, which occur in association with reduced effort-based sucrose consumption, were reversed by administration of the dopamine precursor levodopa (L-DOPA) [15, 16]. As a growing body of literature also demonstrates relationships between increased endogenous inflammation, alterations in activity of and FC within reward circuits, and anhedonia [17–21], understanding potential dopaminergic mechanisms and therapeutic implications is important considering substantial evidence of treatment resistance in MDD with increased inflammation [22–25].

We previously reported that increased plasma C-reactive protein (CRP) concentrations, as well as inflammatory cytokines and their soluble receptors, were associated with low resting-state (rs)FC between the left ventral striatum (VS) and ventromedial prefrontal cortex (vmPFC), key structures of classic reward circuitry that receive mesolimbic and mesocortical dopamine input [26], that correlated with anhedonia in medically stable and unmedicated MDD patients [19]. Relationships between inflammation and low VS-vmPFC rsFC in association with symptoms of anhedonia were observed using both seed-to-voxel-wise and targeted seed-to-ROI approaches [19], corroborated by network analyses [27], and replicated in a sample of highly-traumatized women [28]. Relationships between endogenous inflammation and functional changes in reward circuits were further supported by reduced striatal activation during reward anticipation in depressed patients with higher CRP and other inflammatory markers [20, 21]. Despite evidence of low dopamine function in MDD [29–31] and preferential response to dopaminergic antidepressants in MDD patients with higher levels of CRP [22], whether alterations in reward circuits in MDD patients with higher inflammation and symptoms of anhedonia involve dopaminergic mechanisms and may respond to relevant therapies is currently unknown.

This study examined whether pharmacological challenge with L-DOPA could increase FC in VS-vmPFC reward circuitry in medically stable, unmedicated MDD patients with increased inflammation. To increase rigor and reproducibility, and consistent with our goal of not only understanding mechanisms but also establishing a platform for future studies, a targeted a priori method was used to calculate left VS-vmPFC FC (see above) [19, 28, 32]. To further expand translational potential of results, mean concentrations of plasma CRP (which have clinical relevance, test-retest and inter-lab reliability, and correspond to increased levels of other inflammatory markers in both blood and cerebrospinal fluid) [4] assessed during participant screening were used as the primary measure of inflammation. To confirm that patients with higher CRP had increased concentrations of other inflammatory markers, plasma inflammatory cytokines and their soluble receptors were also measured [4, 19, 28].

We hypothesized that patients with higher (but not lower) levels of CRP would respond with higher VS-vmPFC rsFC after L-DOPA with respect to placebo as the primary outcome, and that similar relationships would be observed for task-based (tb)FC during reward anticipation in the monetary incentive delay (MID) task. It was also hypothesized that rsFC after L-DOPA with respect to placebo would correlate with objective assessments of motivation (effort expenditure for rewards task [EEfRT]) and symptoms of anhedonia (Snaith-Hamilton Pleasure Scale [SHAPS]), and that these relationships would depend on levels of CRP.

## Methods

### Participants

Fifty-seven participants (18–65 years) in a current MD episode determined by Structured Clinical Interview for DSM-5 (SCID-5) [33] were enrolled, and 40 participants with available and analyzable fMRI scans after challenge with both L-DOPA and placebo were included herein (see Supplementary Fig. S1, Supplementary Table S1 and Supplement for data quality exclusions). All subjects were tested for drugs of abuse at screening and pre-scans. All patients were free of psychotropic medications or those affecting the immune system at the time of study entry (see Supplement for details). No subjects were removed from treatment for the purposes of this study, and all subjects were monitored for significant worsening and suicidal risk. Patients were medically stable per medical history, physical exam, and laboratory testing. High sensitivity (hs)CRP was assessed over 2–5 screening visits; values >10 mg/L were retested at 2-week intervals to ensure stability and rule out infection. To maximize a range of values for statistical analyses, patients were recruited to represent a range of inflammation levels from low to high as distributed across mean plasma CRP concentrations 0-1, >1-2, >2-3, and >3 mg/L (20–27.5%/group; see Supplement). The study was registered (Clinicaltrials.gov, NCT02513485) and shared (NIMH Data Archive, #2540). All procedures were approved a priori by the Emory University Institutional Review Board. All participants provided written informed consent.

### Study design and participation

**Fig. 1:**
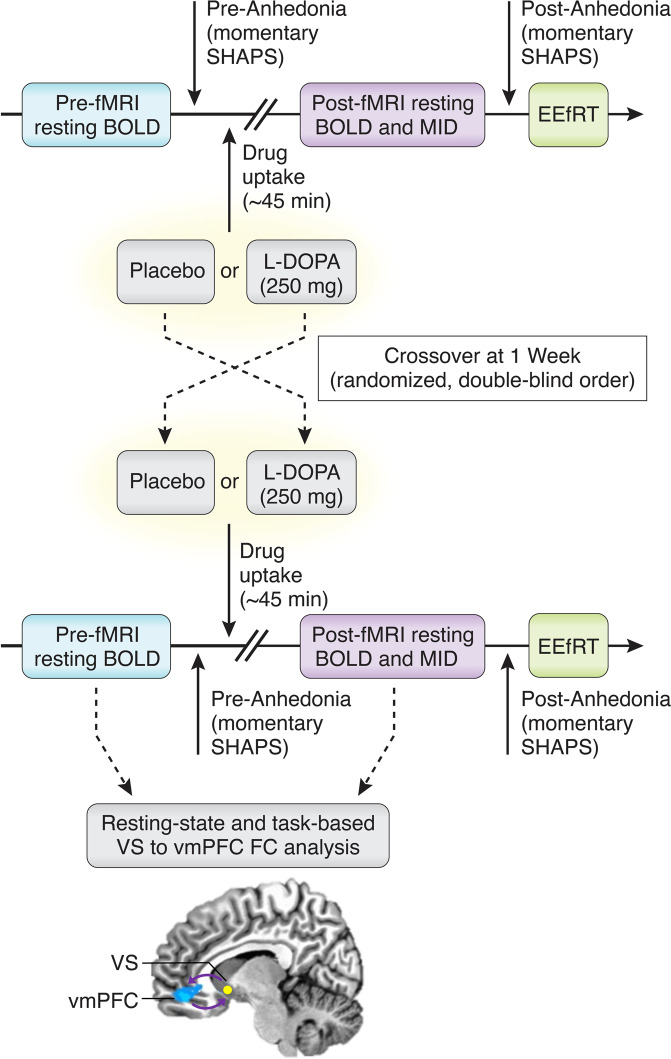
Study design and order of procedures. Abbreviations: BOLD blood oxygen level dependent, EEfRT effort expenditure for rewards task, fMRI functional magnetic resonance imaging, FC functional connectivity, L-DOPA levodopa, MID monetary incentive delay, SHAPS Snaith-Hamilton Pleasure Scale, vmPFC ventromedial prefrontal cortex, VS ventral striatum.

Using a design previously employed in healthy controls that underwent fMRI to assess the effects of L-DOPA on rsFC in corticostriatal circuits [34], patients underwent fMRI and behavioral assessments on separate visits involving acute challenge with L-DOPA (250 mg with 25–50 mg carbidopa; see Supplement for details) and identically encapsulated placebo administered on separate visits spaced ~1-week apart using a double-blind, randomized, cross-over design (Fig. 1). Resting-fMRI and self-reported anhedonia (SHAPS) were collected before and after acute L-DOPA or placebo challenge, and task-fMRI (MID) and objective motivation (EEfRT, outside of scanner) were collected post-L-DOPA or placebo. Although MID and EEfRT have test-retest reliability [35–37], conducting them only post-L-DOPA or placebo administration limited same-day carry-over or practice-effects, fatigue, and promoted task sensitivity (see Supplement and below). Additionally, MID and EEfRT were practiced prior to the first scan visit.

### Behavioral assessments

The 17-item Hamilton Rating Scale for Depression (HAM-D) [38] assessed depression severity; EEfRT, a multi-trial game in which participants choose between two task difficulty levels across varying probabilities of success on each trial in order to obtain monetary rewards [39] that is sensitive to pharmacological manipulation with dopaminergic drugs [40] and inflammation [41], assessed motivation; SHAPS, which has high psychometric validity for assessing the present state of anhedonia [42] and correlates with ecological momentary assessments of negative affect [43], assessed change in hedonic capacity pre and post-L-DOPA and placebo (see Supplement).

### Laboratory measures

Per the mechanistic and translational goals of the study to establish outcomes and recruitment methods for future studies, mean plasma hsCRP concentrations measured during participant screening in the Emory Behavioral Immunology Program were used as the primary outcome variable to classify inflammation levels (see Supplement). To corroborate that patients with mean screening hsCRP concentrations above the identified cut-point for differentiating L-DOPA effects on FC (CRP > 2 mg/L, see Results) had higher concentrations of other inflammatory markers, plasma inflammatory cytokines (IL-1beta, TNF and IL-6) and their soluble receptors were assessed using multiplex immunoassays (R&D Systems, Minneapolis, MN; Supplementary Table S2 and Supplement) [4, 19, 27]. Per prior analyses, cytokine and receptor values were inspected for outliers, a plasma inflammatory composite score was calculated (sum of Z-scores for IL-1beta, TNF, IL-6 and their receptors; see Supplement, Supplementary Fig. S2) [4, 28], and non-normal individual markers were natural log-transformed for statistics [44–46]. Plasma L-DOPA concentrations were measured as described [16, 47, 48] (see Supplement).

### fMRI data acquisition and analyses

Imaging data were acquired at Emory’s Center for Systems Imaging on a Siemens Prisma 3T scanner and 64-channel head coil. A T1-weighted, magnetization prepared rapid gradient echo (MPRAGE) sequence [49] was obtained at 1 mm^3^ resolution. Wakeful resting- and task-fMRI images were acquired by 2D gradient-echo EPI BOLD sequence (see Supplement). Resting-fMRI was acquired both before and after L-DOPA or placebo administration using phase-encoding directions of opposite polarity (anterior-posterior) for distortion correction over ~10 min [50]. Data were analyzed with standard preprocessing protocols in AFNI (http://afni.nimh.nih.gov/; see Supplement). Resting BOLD time series were additionally processed to minimize artifacts from head motion, respiration, cardiac pulsation, and hardware using ANATICOR method [51] via motion/outlier censoring (aka scrubbing), nuisance regression, and band pass filtering (0.009 Hz < f < 0.08 Hz; see Supplement for signal censoring for resting- and task-fMRI). Individuals’ 4D fMRI data were spatially normalized into standard Montreal Neurological Institute (MNI) space. For both resting- and task-fMRI (see MID below) analyses, left-VS (see Supplement for justification and exploratory results for right-VS) to vmPFC FC was assessed using a targeted, a priori seed-to-ROI approach. Subject-level Fisher’s normalized Z-scores were extracted for FC between a 3 mm^3^ radius spherical seed centered on left-VS (including nucleus accumbens) [52, 53] and vmPFC ROI previously associated with neural activation to receipt of reward in neuroimaging meta-analysis [54] and used in our previous work [19, 27, 28, 55]. This unbiased, targeted approach was chosen to increase rigor, reproducibility, and potential application of results to future trials (see Supplement).

### MID task

VS-vmPFC tbFC during reward anticipation was assessed from two functional scan runs of 70 trials each over ~20 min [56–58]. The anticipatory delay (~4000 ms) occurred after a pseudo-randomly presented cue informed participants whether a given trial allowed them to win or lose money (reward: +$; loss: −$; no incentive: 0$; averaging ~$2) but prior to the target stimulus. Monetary outcome depended on a simple button-press reaction in response to a visual target stimulus. Task-based FC during each anticipation condition was assessed using beta-series correlation [59], a powerful and sensitive method to estimate task-modulated FC [60, 61] that has been used with MID [56, 58]. The beta-series were derived from a design matrix with separate regressors for each trial (see above and Supplement). The beta of interest was reward anticipation during stimulus cue of monetary gain versus neutral.

### Statistics

Patient characteristics were summarized using mean and standard deviation (SD) for continuous and percent for categorical variables. In patients where both pre- and post-L-DOPA and placebo rsFC date were available and analyzable (*n* = 31; see Results and Supplement), response (change in VS-vmPFC rsFC; post minus pre) to L-DOPA with respect to placebo was first examined by repeated-measures general linear model with plasma CRP concentration as a continuous variable, both with and without clinical and demographic covariates that may confound relationships between inflammation and the brain and behavior (age, sex, race and body mass index [BMI]), as well as study-related variables such as plasma L-DOPA concentrations, order of treatment, etc. (see Supplement for details) in separate analyses. To further interpret a significant treatment by CRP interaction, response to L-DOPA and placebo was plotted across a range of CRP levels to identify a potentially sensitive cut-point. To assess whether rsFC responses to L-DOPA with respect to placebo were significantly different in patients above versus below the identified CRP cut-point (>2 mg/L, see Results and Supplement), and consistent with analyses for variables where only post-challenge data were available, generalized estimating equations (GEE) with identity link function and interchangeable working correlation matrix were implemented both with and without covariates. Linear regression models probed relationships between rsFC response to L-DOPA and CRP (as a continuous variable) and the inflammatory composite score. Backward and forward linear regression using the same criteria for entry and removal were employed to identify which marker (from the composite score) was the most significant predictor. Logistic regression and receiver operating characteristic (ROC) analyses also examined whether rsFC response to L-DOPA (change in rsFC Z-scores, post minus pre) was able to classify patients as having CRP > 2 mg/L versus other potential cut-points (CRP 1 or 3 mg/L). GEE was further utilized to determine whether patients with higher CRP levels had higher VS-vmPFC rsFC after L-DOPA with respect to placebo in the full sample with analyzable post-challenge data (*n* = 40; see below and Supplement). Similar analyses examined potential effects of CRP level after L-DOPA with respect to placebo on tbFC during reward anticipation (win>neutral, MID), motivation (proportion of high effort choices, EEfRT), and anhedonia response (SHAPS scores, post minus pre), with and without covariates. Relationships between rsFC and behavior were examined by linear regression in models including treatment, CRP level and the effects of their interaction. See detailed *Power Calculation* in Supplement. All tests of significance were two-tailed with α < 0.05, conducted in IBM SPSS Statistics 28.

## Results

Of 56 patients that completed scans including challenge with both placebo and L-DOPA in the parent study (Fig. 1 and Supplementary Fig. S1), the final dataset herein included 40 patients with available and analyzable rsFC data after both L-DOPA and placebo challenge. A subset of these patients also had analyzable rsFC data both before “pre”- and after “post”-L-DOPA and placebo (*n* = 31) that was used to assess response to L-DOPA and placebo as change in FC (Z-scores, post minus pre). Data for tbFC during reward anticipation (in MID) and motivation (EEfRT, outside of scanner) were also available/analyzable in a subset of patients, while anhedonia response to L-DOPA and placebo (SHAPS scores, post minus pre) was available in all subjects (see Supplementary Fig. S1 and Supplement). Demographic, immune, and clinical characteristics were similar between the analyzed and studied sample (see Supplementary Table S1). Characteristics of the 40 analyzed patients are summarized by the identified CRP cut-point (> versus ≤2 mg/L, see Results) in Table 1. Importantly, patents with CRP > 2 mg/L had further evidence of increased peripheral inflammation per the inflammatory composite score for cytokines and their receptors (*t* = −2.69, df = 37, *p* = 0.011; Table 1, Supplementary Fig. S2). Acute L-DOPA administration was generally well-tolerated in that adverse event (AEs) were mild to moderate, anticipated, resolved by the end of study visits, and did not differ between patients with CRP > versus ≤ 2 mg/L (see Supplement). The only non-inflammatory variable higher in patients with CRP > 2 mg/L was BMI, which was controlled along with other covariates (age, sex, race) in all analyses.

**Table 1:** Demographic and clinical variables of the study sample by CRP category.

		CRP ≤ 2 mg/L (*N* = 21)	CRP > 2 mg/L (*N* = 19)	*p* value
Age	Mean (SD)	34.0 (10.6)	38.5 (11.4)	0.206^a^
Sex	Male, *N* (%)	9 (42.9)	3 (15.8)	0.089^b^
Race	White, *N* (%)	13 (61.9)	11 (57.9)	0.967^c^
	Black, *N* (%)	7 (33.3)	7 (36.8)	
	Asian, *N* (%)	1 (4.8)	1 (5.3)	
BMI	Mean (SD)	26.0 (4.3)	31.2 (5.9)	**0.003**^a^
CRP (mg/L)	Median (IQR)	1.0 (1.1)	3.63 (1.4)	**<0.001**^d^
Inflammatory Score^f^	Mean (SD)	−1.2 (2.5)	1.2 (3.1)	**0.011**^a^
HAM-D	Mean (SD)	21.5 (3.2)	22.0 (3.9)	0.677^a^
IDS-SR	Mean (SD)	34.5 (9.4)	39.2 (5.7)	0.069^a^
EEfRT (proportion hard choices per % probability win)^g^				
12%, Post-Placebo	Mean (SD)	0.14 (0.22)	0.08 (0.12)	0.309^e^
12%, Post-L-DOPA	Mean (SD)	0.14 (0.22)	0.09 (0.16)	0.487^e^
50%, Post-Placebo	Mean (SD)	0.33 (0.24)	0.23 (0.22)	0.202^e^
50%, Post-L-DOPA	Mean (SD)	0.34 (0.25)	0.23 (0.26)	0.218^e^
88%, Post-Placebo	Mean (SD)	0.58 (0.18)	0.51 (0.33)	0.428^e^
88%, Post-L-DOPA	Mean (SD)	0.52 (0.21)	0.53 (0.32)	0.845^e^
Anhedonia (momentary SHAPS score)				
SHAPS Pre-Placebo	Mean (SD)	4.0 (3.5)	5.4 (4.3)	0.259^e^
SHAPS Post-Placebo	Mean (SD)	3.1 (3.3)	5.3 (4.4)	0.091^e^
SHAPS Pre-L-DOPA	Mean (SD)	4.1 (3.1)	4.9 (4.2)	0.466^e^
SHAPS Post-L-DOPA	Mean (SD)	3.6 (3.4)	3.9 (4.4)	0.762^e^
Plasma L-DOPA (nM)	Median (IQR)	5650 (6085)	7370 (6130)	0.215^d^
Plasma DA (nM)	Median (IQR)	9.72 (5.84)	9.08 (6.86)	0.893^d^

*CRP* C-reactive protein, *BMI* body mass index, *DA* dopamine, *EEfRT* Effort Expenditure for Rewards Task, *HAM-D* Hamilton Depression Rating Scale, *L-DOPA* levodopa, *SHAPS* Snaith-Hamilton Pleasure Scale, *SD* standard deviation, *IQR* interquartile range.

Bold text indicates *p* < 0.05.

^a^T-test.

^b^Fisher’s exact test.

^c^Pearson’s Chi-squared test.

^d^Kruskal–Wallis rank sum test.

^e^Multivariate linear ANOVA.

^f^*n* = 20 for CRP ≤ 2 mg/L group.

^g^*n* = 17 for CRP > 2 mg/L group.

### VS-vmPFC FC response to L-DOPA with respect to placebo: role of plasma CRP

#### rsFC analyzed pre- and post-L-DOPA and placebo

**Fig. 2:**
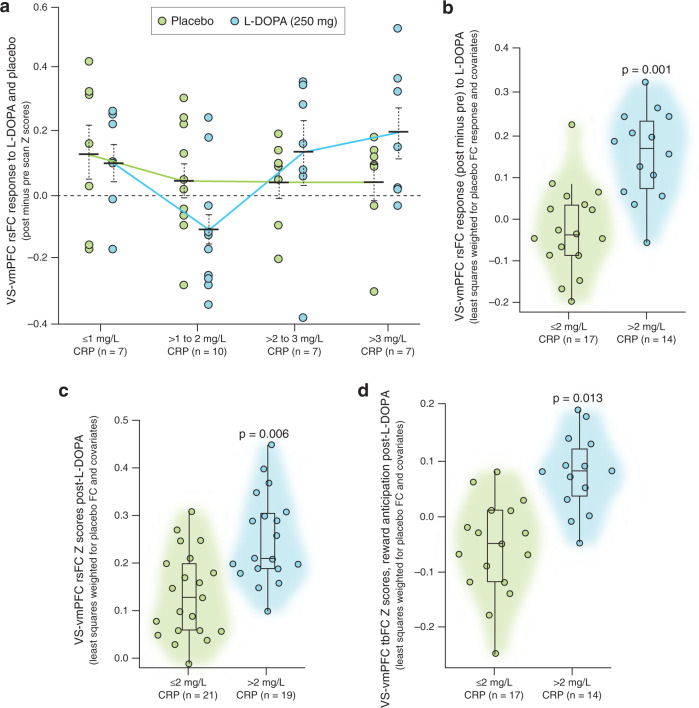
Depressed patients with plasma CRP > versus ≤ 2 mg/L had higher VS-vmPFC FC after L-DOPA with respect to placebo. In patients with VS-vmPFC rsFC available both pre- and post-L-DOPA and placebo (*n* = 31), rsFC responses (post minus pre) across a range of plasma CRP concentrations revealed that only patients with CRP > 2 mg/L had mean (black bars) positive responses (FC change > 0) after L-DOPA but not placebo (**a**). The rsFC response to L-DOPA was significantly higher in patients with CRP > versus ≤ 2 mg/L when controlling for response to placebo (**b**). In the full sample with analyzable rsFC data after both L-DOPA and placebo (*n* = 40), VS-vmPFC rsFC after L-DOPA with respect to placebo was also higher in patients with CRP > versus ≤ 2 mg/L (**c**). Similar relationships were observed during reward anticipation in the MID whereby VS-vmPFC tbFC after gain versus neutral cues was higher after L-DOPA with respect to placebo in patients with CRP > 2 mg/L (**d**). Individual subject data over violin plots with median and IQR. Abbreviations: CRP C-reactive protein, FC functional connectivity, L-DOPA levodopa, rs resting-state, tb task-based, vmPFC ventromedial prefrontal cortex, VS ventral striatum, IQR interquartile range.

Data from patients with analyzable rsFC data both pre- and post-L-DOPA and placebo (*n* = 31) were used to determine relationships between response (change in rsFC Z-scores, post minus pre) to L-DOPA and placebo and inflammatory markers, including determining a sensitive cut-point for CRP to classify patients exhibiting a positive response to L-DOPA, as well as to assess stability of targeted VS-vmPFC rsFC, which served as the primary outcome. Regarding stability, VS-vmPFC rsFC Z-scores during “pre” scans (before administration of study medication) were highly stable across visits (mean 0.16 ±~0.17 on both visits, paired *t* = −0.03, df = 29, *p* = 0.997). While no effect of treatment alone (L-DOPA versus placebo) on VS-vmPFC rsFC was observed in a repeated-measures linear model (*p* = 0.952), an interaction with CRP as a continuous variable indicated that response to L-DOPA depended on levels of inflammation (F[1,25] = 4.4, *p* = 0.046 with, and F[1,29] = 6.9, *p* = 0.022 without, the above-mentioned covariates - age, sex, race and BMI). Examination of rsFC responses to L-DOPA and placebo across a range of plasma CRP concentrations (0-1, >1-2, >2-3, and >3 mg/L) showed that only patients with CRP > 2 mg/L had a positive response to L-DOPA (mean change in rsFC greater than both 0 and the response to placebo; Fig. 2a). To further interpret these findings, and consistent with analyses for outcomes where only post- assessments were available and response (post minus pre) could not be assessed, GEE models were employed. These results revealed that VS-vmPFC rsFC response to L-DOPA was significantly higher in patients with CRP > versus ≤ 2 mg/L when controlling for response to placebo (Fig. 2b), *B* = 0.23, SE(B) = 0.07, *p* = 0.001 with and *B* = 0.19, SE(B) = 0.07, *p* = 0.008 without covariates. Of note, VS-vmPFC rsFC response to L-DOPA correlated with both CRP concentrations (*r* = 0.40, df = 28, *p* = 0.027) and inflammatory composite scores (*r* = 0.37, df = 27, *p* = 0.047) in linear models controlling for placebo response, and IL-6 was the only significant predictor in multiple linear regression with backward and forward selection using the same criteria for entry and removal that included covariates (*r* = 0.44, *p* = 0.015). Finally, logistic regression and ROC analyses lent further support for CRP > 2 mg/L as a sensitive cut-point for response to L-DOPA (see Supplementary Fig. S3).

#### rsFC and tbFC during reward anticipation analyzed post-L-DOPA and placebo

Consistent with the above findings that patients with CRP > 2 versus ≤ 2 mg/L had a positive and significantly higher VS-vmPFC rsFC response (post minus pre, *n* = 31) to L-DOPA with respect to placebo, VS-vmPFC rsFC post-L-DOPA with respect to placebo was also higher in patients with CRP > versus ≤ 2 mg/L in the full sample with analyzable rsFC data after both L-DOPA and placebo (*n* = 40, Fig. 2c), *B* = 0.15, SE(B) = 0.05, *p* = 0.006 in GEE models with and *B* = 0.10, SE(B) = 0.05, *p* = 0.031 without covariates. Similarly, in subjects with analyzable tbFC from MID data that was collected after L-DOPA and placebo, relationships were observed during reward anticipation whereby VS-vmPFC tbFC after gain versus neutral cues was higher after L-DOPA with respect to placebo in patients with CRP > 2 mg/L (Fig. 2d) *B* = 0.15, SE(B) = 0.06, *p* = 0.013 in GEE models with and *B* = 0.15, SE(B) = 0.05, *p* < 0.001 without covariates. Of note, all above-reported relationships between CRP level and VS-vmPFC rs or tbFC after L-DOPA challenge remained significant when controlling for study-related variables including plasma L-DOPA concentrations and treatment order (see Supplement for details).

### Change in anhedonia negatively correlated with VS-vmPFC rsFC after L-DOPA in patients with CRP > 2 mg/L

**Fig. 3:**
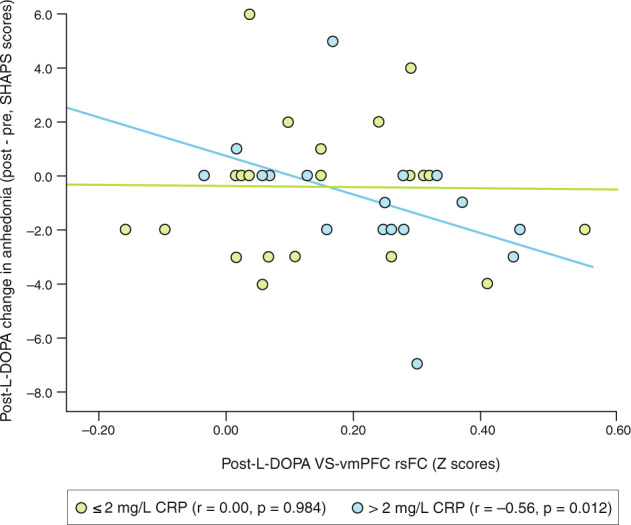
Change in anhedonia scores negatively correlated with VS-vmPFC rsFC after L-DOPA challenge only in patients with CRP > 2 mg/L. An interaction was observed for treatment and CRP level on the relationship between rsFC and change in anhedonia (SHAPS scores, post minus pre), whereby a decrease in anhedonia was correlated with VS-vmPFC rsFC after L-DOPA but only in patients with CRP > 2 mg/L. CRP C-reactive protein, FC functional connectivity, L-DOPA levodopa, SHAPS Snaith-Hamilton Pleasure Scale, rs resting state, vmPFC ventromedial prefrontal cortex, VS ventral striatum.

Motivation (hard-task choices on EEfRT) and anhedonia (SHAPS scores) were not significantly affected by L-DOPA alone or in relation to CRP level (all *p* > 0.152; see means in Table 1 and Supplement for details). Motivation (EEfRT, proportion of hard choices in 50 and 88% reward probability conditions) correlated with VS-vmPFC rsFC (*r* = 0.24–0.29, *p* < 0.05), but did not depend on treatment or CRP (Table 2). However, as anticipated, an interaction was observed for treatment and CRP level on the relationship between VS-vmPFC rsFC and anhedonia response (SHAPS scores, post minus pre; *r* = −0.29, df = 74, *p* = 0.011; Table 2), whereby a decrease in anhedonia was correlated with VS-vmPFC rsFC after L-DOPA only in patients with CRP > 2 mg/L (Fig. 3; *r* = −0.56, df = 17, *p* = 0.012). This interaction remained significant when controlling for covariates (*r* = −0.24, df = 70, *p* = 0.041) as well as study-related variables like L-DOPA concentrations and treatment order (see Supplement).

**Table 2:** Relationships between VS-vmPFC resting-state (rs)FC and behaviors related to reduced motivation and anhedonia after placebo and L-DOPA.

Dependent: EEfRT - proportion of hard task choices at 88% probability of winning	
Independent: Variables	*r*, *p* value
VS-vmPFC rsFC	**0.24, 0.039**
VS-vmPFC rsFC	**0.24, 0.041**
Treatment (L-DOPA or Placebo)	−0.02, 0.885
CRP (> or ≤2 mg/L)	−0.07, 0.557
VS-vmPFC rsFC	0.16, 0.177
Treatment (L-DOPA or Placebo)	−0.04, 0.722
CRP (> or ≤2 mg/L)	−0.10, 0.421
FC × Treatment × CRP Interaction	0.07, 0.567
Dependent: EEfRT - proportion of hard task choices at 50% probability of winning	
Independent: Variables	*r*, *p* value
VS-vmPFC rsFC	**0.29, 0.048**
VS-vmPFC rsFC	**0.25, 0.029**
Treatment (L-DOPA or Placebo)	0.05, 0.687
CRP (> or ≤2 mg/L)	**−0.23, 0.046**
VS-vmPFC rsFC	0.20, 0.084
Treatment (L-DOPA or Placebo)	0.04, 0.744
CRP (> or ≤2 mg/L)	−0.15, 0.210
FC × Treatment × CRP Interaction	0.01, 0.915
Dependent: EEfRT - proportion of hard task choices at 12% probability of winning	
Independent: Variables	*r*, *p* value
VS-vmPFC rsFC	0.14, 0.232
VS-vmPFC rsFC	0.16, 0.188
Treatment (L-DOPA or Placebo)	0.04, 0.770
CRP (> or ≤2 mg/L)	−0.16, 0.188
VS-vmPFC rsFC	0.19, 0.115
Treatment (L-DOPA or Placebo)	0.07, 0.541
CRP (> or ≤2 mg/L)	−0.01, 0.954
FC × Treatment × CRP Interaction	−0.10, 0.379
Dependent: Anhedonia response (change in SHAPS score, post minus pre)	
Independent: Variables/Covariates	*r*, *p* value
VS-vmPFC rsFC	−0.05, 0.671
VS-vmPFC rsFC	−0.06, 0.581
Treatment (L-DOPA or Placebo)	−0.10, 0.404
CRP (> or ≤2 mg/L)	0.03, 0.803
VS-vmPFC rsFC	0.24, 0.034
Treatment (L-DOPA or Placebo)	0.12, 0.313
CRP (> or ≤2 mg/L)	0.25, 0.031
FC × Treatment × CRP interaction	**−0.29, 0.011**

Correlation coefficient (*r*) and *p* values reported for the association between L-DOPA resting-state FC and behavioral measures.

*VS-vmPFC FC* functional connectivity Z-scores between ventral striatum (VS) and ventromedial prefrontal cortex (vmPFC), *L-DOPA* levodopa, *CRP* C-reactive protein, *BMI* body-mass index, *EEfRT* effort expenditure task, *SHAPS* Snaith-Hamilton Pleasure Scale.

Bold text indicates *p* < 0.05.

## Discussion

The primary outcome of rsFC in a classic VS-vmPFC reward circuit was higher in response to acute challenge with L-DOPA compared to placebo in patients with plasma CRP concentrations > but not ≤ 2 mg/L. Whereas patients with ≤1 mg/L CRP had similar moderate rsFC responses to L-DOPA as to placebo, those with >1 to 2 mg/L CRP had on average reduced rsFC responses to L-DOPA with respect to placebo (Fig. 2a). While this likely contributed to group differences in rsFC responses in patients with CRP > versus ≤2 mg/L, it should be noted that when analyzed separately, a medium to large effect size was seen in patients with CRP > 2 mg/L for the rsFC response to L-DOPA with respect to placebo (Cohen’s dz=0.61), an effect that was significant in patients with CRP > (*p* = 0.034) but not ≤ (*p* = 0.412) 2 mg/L in linear models including covariates. Thus, L-DOPA increased rsFC with respect to placebo in patients with CRP > 2 mg/L irrespective of responses in patients with CRP ≤2 mg/L. This relationship was also observed in task-modulated VS-vmPFC FC during reward anticipation (MID, gain>neutral). Moreover, an interaction was observed whereby reduced anhedonia in response to L-DOPA correlated with post-L-DOPA rsFC only in patients with CRP > 2 mg/L. These results suggest that our targeted, a priori method for assessing FC in reward circuitry that has been reproducibly associated with both increased inflammation and anhedonia [19, 28, 32] is a potentially modifiable brain biomarker to assess target engagement in relation to behavioral efficacy of treatments to reverse the impact of inflammation on the brain in MDD. In this regard, these findings also suggest that MDD patients with higher CRP may have low dopamine availability and potential for therapeutic benefit from therapies with dopaminergic activity [22].

The identified cut-point of CRP > 2 mg/L for differential FC response to L-DOPA is consistent with median concentrations of CRP in this and prior studies using similar recruitment methods [4, 19], the middle of moderate risk CRP range per American Heart Association [62], and was used as enrollment criteria in a recent trial for IL-1 antagonism in atherosclerotic disease [63]. While antidepressant benefit of anti-cytokine therapies has been limited to patients with higher levels of CRP (>3–5 mg/L) in samples of treatment-resistant depressed patients (who exhibit higher median levels of CRP) [64–66], differential responses to a bupropion add-on in patients who failed to respond to a selective serotonin reuptake inhibitor were seen at CRP > 1 mg/L [22]. While blockade of inflammation with anti-cytokine therapies may exert efficacy through effects on dopamine and reward circuitry, particularly considering that anhedonia-related symptoms are most improved [64–66], risk of inhibiting beneficial effects of innate immune signaling on other neurobiological pathways like myelination and concern for immunosuppression limit translation of current cytokine antagonists for depression [67, 68]. Nevertheless, whether existing antidepressants like bupropion have benefit as monotherapy in MDD with higher levels of CRP warrants investigation, as per our ongoing study collecting preliminary evidence of the efficacy of bupropion versus escitalopram to increase VS-vmPFC rs and/or tbFC in association with reduced anhedonia in MDD patients with CRP > 2 mg/L (NCT04352101). While use of L-DOPA, the precursor for dopamine with known pharmacology and pharmacodynamics in the brain, as a challenge in this study was well-matched to mechanisms by which inflammation may reduce dopamine availability, it may also have potential antidepressant efficacy in select patients. Indeed, a recent study administering L-DOPA to older depressed patients (a population with reliably increased inflammation) with motor slowing showed improvements in both motor speed and depression severity after sub-chronic treatment with similar doses [69]. Inflammation-sensitive regions of VS and vmPFC are critically modulated by mesolimbic and mesocortical dopamine [1, 70], yet it should be noted that inflammation can affect numerous neurotransmitter and neurobiological substrates like glutamate that may also influence FC within interconnected regions of striatum and PFC in association with transdiagnostic symptoms like anhedonia, and which may serve as additional therapeutic targets for patients with increased inflammation [71, 72].

A primary limitation included small sample size from lack of quality FC data in all participants (see Supplement for discussion), yet with no difference in demographic, immune or clinical variables between analyzed and studied samples. L-DOPA also had a high rate of expected AEs, possibly due to acute effects in naïve patients as 4-weeks of L-DOPA was well-tolerated in older depressed patients [69]. While the study design did allow examination of stability of VS-vmPFC rsFC as a primary outcome, it did not permit examination of whether CRP concentrations assigned at screening were stable throughout the study as blood draws were not conducted before assessments to reduce burden and potential influence of stress on study outcomes. However, higher levels of cytokines and their soluble receptors (Table 1) and the classification of patients as having CRP > versus **≤** 2 mg/L based on their rsFC responses to L-DOPA (Supplementary Fig. S3) support that our screening methods (consistent with AHA guidelines for establishing CRP stability) for classification at this cut-point reflected both higher inflammation in the periphery and differential neurobiological responses to L-DOPA, and may be used to screen and enroll patients with CRP > 2 mg/L in future studies. While significant effects on behavior after a single L-DOPA administration was not anticipated, we did expect relationships between behavior and FC whereby changes in behavior would be seen in higher inflammation patients with the greatest FC response to L-DOPA, which was only observed for anhedonia. While lack of a significant L-DOPA by CRP interaction on motivation in relation to FC was not consistent with hypotheses, a single dose of L-DOPA may not have been as strong as other manipulations (e.g., dopamine release with d-amphetamine) [36, 37] to overcome fatigue/practice-effects from repeat EEfRT administration at the end of visits (~2–3 h post-L-DOPA). Future work will examine whether chronic increases in dopamine availability with repeated L-DOPA administration have sustained effects on reward circuits and/or downstream behaviors, including our ongoing study that aims to determine the best dose of L-DOPA to increase FC and improve behavior over time in a larger sample of patients with CRP > 2 mg/L (NCT04723147). Follow-up analyses of this study will also explore relationships between the acute effects of L-DOPA, CRP and behavior in other circuits linked to high inflammation such as corticostriatal FC involving dorsal striatum in relation to psychomotor behaviors [19, 27]. Despite limitations, promising results suggest targeted VS-vmPFC FC as a modifiable biomarker of inflammation effects on the brain that can be applied to longitudinal studies examining novel therapeutic strategies including dopaminergic agents in MDD or other patients with increased CRP.

## Supplementary information

Supplemental Information
