# Correction to: Functional connectivity in reward circuitry and symptoms of anhedonia as therapeutic targets in depression with high inflammation: evidence from a dopamine challenge study

**DOI:** 10.1038/s41380-022-01754-w

**Published:** 2022-09-05

**Authors:** Mandakh Bekhbat, Zhihao Li, Namrataa D. Mehta, Michael T. Treadway, Michael J. Lucido, Bobbi J. Woolwine, Ebrahim Haroon, Andrew H. Miller, Jennifer C. Felger

**Affiliations:** 1 Department of Psychiatry and Behavioral Sciences, Emory University, Atlanta, GA 30322 USA; 2 School of Psychology and Sociology, Shenzhen University, Shenzhen, 518060 Guangdong Sheng China; 3 Neuroscience Graduate Program, Graduate Division of Biological and Biomedical Sciences, Emory University, Atlanta, GA 30322 USA; 4 Department of Psychology, Emory University, Atlanta, GA 30322 USA; 5 The Winship Cancer Institute, Emory University, Atlanta, GA 30322 USA

**Keywords:** Neuroscience, Depression

Correction to: *Molecular Psychiatry* 10.1038/s41380-022-01715-3, published online 04 August 2022

The article “Functional connectivity in reward circuitry and symptoms of anhedonia as therapeutic targets in depression with high inflammation: evidence from a dopamine challenge study”, written by Mandakh Bekhbat, Zhihao Li, Namrataa D. Mehta, Michael T. Treadway, Michael J. Lucido, Bobbi J. Woolwine, Ebrahim Haroon, Andrew H. Miller, Jennifer C. Felger, was originally published electronically on the publisher’s internet portal on 4 August 2022 without open access. With the author(s)’ decision to opt for Open Choice the copyright of the article changed on 14 August 2022 to © The Authors 2022 and the article is forthwith distributed under a Creative Commons Attribution 4.0 International License, which permits use, sharing, adaptation, distribution and reproduction in any medium or format, as long as you give appropriate credit to the original author(s) and the source, provide a link to the Creative Commons licence, and indicate if changes were made. The images or other third party material in this article are included in the article’s Creative Commons licence, unless indicated otherwise in a credit line to the material. If material is not included in the article’s Creative Commons licence and your intended use is not permitted by statutory regulation or exceeds the permitted use, you will need to obtain permission directly from the copyright holder. To view a copy of this licence, visit http://creativecommons.org/licenses/by/4.0/.

