# Supplementary material for: Functional connectivity in reward circuitry and symptoms of anhedonia as therapeutic targets in depression with high inflammation: evidence from a dopamine challenge study
Source: Mol Psychiatry. 2022 Aug 4;27(10):4113–21. doi: 10.1038/s41380-022-01715-3 (PMC9718669; doi:10.1038/s41380-022-01715-3)
Supplement: Supplementary file 1 — Supplemental Information [file 41380_2022_1715_MOESM1_ESM.pdf]

SUPPLEMENTAL INFORMATION FOR:

Functional connectivity in reward circuitry and symptoms of anhedonia as therapeutic targets in depression with high inflammation: evidence from a dopamine challenge study

Bekhbat, et al., *Molecular Psychiatry*

METHODS

Screening and enrollment. Medically stable male and female adult (18-65) MDD outpatients were recruited from the community by social media advertising, postings on Emory websites, and clinicaltrials.gov, and screened for participation in ongoing studies of depression in the Emory Behavioral Immunology Program between fall 2015 and spring 2020. Sixty qualified patients were identified for the parent study before it was halted and then closed during the COVID-19 pandemic. Enrolled patients had a primary diagnosis of MDD (or bipolar disorder current episode depressed; n=1) and met criteria for a current major depressive episode without psychosis according to DSM-5 criteria (using SCID-5) and with a symptom severity score of ≥16 on the Quick Inventory of Depressive Symptomatology-Self Report (QIDS-SR 16). All patients were free of psychotropics for at least 4 weeks (8 weeks for fluoxetine) at the time of study entry; *no subjects were removed from treatment for this study*. All subjects were monitored for significant worsening and suicidal risk. To be included, subjects must not have demonstrated active suicidal intent or plan, must have scored 2 or below on the HAM-D-17 suicide item (item #3), and must have made no prior suicide attempt within six months of screening. Enrolled subjects had no lifetime history of the following: schizophrenia, schizoaffective disorder, any other (non-mood) psychotic disorder, intellectual disability, amnestic disorder, dementia or Mini-Mental State Exam (MMSE) <28 (clinically significant cognitive dysfunction), or delirium; and substance dependence or abuse within the past 6 months (except nicotine). Patients with contra-indications to MRI including embedded metallic objects, prosthetics or implants made of paramagnetic metals or ferromagnetic alloys, aneurysmal clips and/or a prior history of claustrophobia were excluded. Subjects were excluded if urine toxicology screen was positive for drugs of abuse. The presence of comorbid dysthymia and/or an anxiety-related disorder including generalized anxiety disorder, panic disorder, post-traumatic stress disorder and social phobia did not disqualify subjects from enrollment as long as major depression was deemed to be the predominant diagnosis as determined by SCID-V and board-certified clinician assessment. Subjects with antisocial personality disorder were excluded. Current obsessive-compulsive disorder was exclusionary only if impacting daily functioning, as assessed by clinical interview. Patients with an active eating disorder were also excluded but patients with binge eating disorder in whom binging is clearly associated with worsening of mood symptoms were included. Medications for other medical conditions were allowed as dictated by the patients' treating physicians; patients were required to be medically stable as determined by medical history, physical exam, and laboratory testing. To ensure inflammatory measurements are not confounded by underlying medical conditions, patients with history of or current heart disease, head trauma, epilepsy, stroke, malignancy not in recovery, autoimmune disorder, organ transplant, inflammatory bowel disease, unstable cardiovascular, endocrinologic, hematologic, autoimmune, hepatic, renal, or neurologic disease (determined by physical examination and laboratory testing), chronic infection (including hepatitis B or C or HIV), or current positive pregnancy test or lactation, were excluded. Participants with evidence of active infections were excluded until medically stable. High sensitivity (hs)CRP was assessed on 2-5 screening visits over a period of weeks (on average ~1.5 months but no more than 90 days before the first scan visit and with values within 25%) with any CRP value >10 mg/L retested at 2-week intervals to ensure stability and, along with the

clinical and laboratory assessments described above, rule out infection. To maximize a range of values for statistical analyses, patients were recruited to represent a full range of inflammation from low to high so that participants were approximately equally distributed across a range of mean plasma CRP concentrations from 0-1, >1-2, >2-3, and >3 mg/L (20 to 27.5% per group). Contraindications to L-DOPA including history of narrow-angle glaucoma, melanoma, gastric and/or duodenal ulcers, bleeding disorders, or debilitating migraine (>6 headaches per month) were also excluded. The study was registered at clinicaltrials.gov (NCT02513485) and shared on NIMH Data Archive (#2540). All procedures were approved *a priori* by the Institutional Review Board of Emory University. All participants provided written informed consent.

Study participation. Of the 60 patients that qualified, three experienced anxiety or claustrophobia during the first scan (despite screening for claustrophobia, per above), and one participant experienced moderate nausea and vomiting after receiving the study medication at the first visit, and thus withdrew from further study participation (**Figure S1**). Therefore, 56 participants completed both study visits including challenge with L-DOPA and placebo on separate weeks in a double-blind, randomized order (**Figures 1** and **S1**, and **Table S1**). Resting-state fMRI and self-reported anhedonia (SHAPS) were collected before and after acute challenge with L-DOPA or placebo, and task-based fMRI (Monetary Incentive Delay [MID]) and objective motivation (Effort Expenditure for Reward Task [EEfRT]) were assessed after L-DOPA or placebo (**Figure 1**). Although both MID and EEfRT have established test-retest reliability[1-3], they were conducted only after L-DOPA or placebo administration to avoid potential same-day carry over or practice effects, limit fatigue, and promote task sensitivity. The MID and EEfRT were additionally practiced ~1-week prior to the first study visit. A final dataset of patients with analyzable resting-state fMRI scans (see "*MRI procedures"* and "*fMRI data acquisition and analyses"* sections below for discussion and criteria for data quality and censoring) after both L-DOPA and placebo challenge were available from 40 participants (**Figure S1**), and a subset of these patients (n=31) also had analyzable pre- and post-L-DOPA and placebo resting-state fMRI to assess response (change in FC: post minus pre) to L-DOPA and placebo. Post-L-DOPA and placebo analyzable data were also available from 31 participants to assess FC during reward anticipation in the MID, and 38 patients to assess motivation outside of the scanner by EEfRT (**Figure S1**). All patients completed pre-post SHAPS assessment of change in anhedonia symptoms to analyze in relation to neuroimaging data at both study visits, except for a missing question from one participant at the second study visit prior to administration of study medication. Thus, the total score for this subject at their "pre" timepoint was replaced with the total SHAPS score at the "pre" timepoint from the prior visit.

Study medication: dosing strategy and management of side effects. Acute administration of L-DOPA can cause mild to moderate peripheral side effects such as nausea or vomiting, dizziness, and headache. To reduce these symptoms and improve uptake of L-DOPA for conversion to dopamine in the brain, carbidopa is co-administered. To determine an acute dose of L-DOPA-carbidopa that would be sufficient to increase VS-vmPFC FC in patients with high inflammation, we tested two common clinical doses in range with those previously used in fMRI studies (100-300 mg)[4-7] in 4 medically stable MDD patients (1 male, 3 female) recruited to have CRP >2 mg/L that were imaged before and ~45 min after acute administration of L-DOPA (corresponding to peak plasma levels at 30-60 min)[8, 9]. Two patients received L-DOPA-carbidopa 100/25 mg and two received 250/25 mg. Individual patient level VS-vmPFC FC before and after L-DOPA administration was calculated as described below[10], and we found that L-DOPA increased connectivity in 3 of 4 patients, with a trend toward significance ($t=-2.04$, $df=3$, $p=0.13$, effect size $d=1.02$), despite the small sample size. Patients receiving the 250/25 mg had ~2-fold greater increase in connectivity compared to those given 100 mg, as well as 64% higher plasma L-DOPA concentrations. Thus, 250/25 mg was chosen for use in this study, and was encapsulated and

administered with an identical placebo. Because protein can affect absorption of L-DOPA into the brain[11], patients were asked not to eat before their scans[4], which were scheduled to start in the morning (~10 AM ±2 hours). Patients were given crackers or a similar low-protein snack prior to taking the study medication, and antacid was offered after the last scan before patients received lunch. Due to potential for L-DOPA to cause orthostatic hypotension, blood pressure was also monitored before administration of study medication, ~30 min after administration of study medication, and after the final scan. While the first 10 patients (4 from the pilot study and 6 in the parent study) did not report any adverse events (AEs) after L-DOPA administration, an increased percentage of subject experienced AEs starting in 2016 (see below). In consultation with a neurologist on the Data and Safety Monitoring Board (DSMB) for the study, the carbidopa dose was increased from 25 to 50 mg in January 2017. Of the 40 patients analyzed in this study, 13 received L-DOPA-carbidopa 250/25 and 27 received 250/50 mg. As plasma L-DOPA concentrations were ~40% lower ($p<0.05$) in patients receiving 50 versus 25 mg carbidopa, L-DOPA concentrations were controlled in all analyses (see below).

Behavioral assessments. Anhedonia: The Snaith-Hamilton Pleasure Scale (SHAPS), which has high psychometric validity for assessing the present state of anhedonia[12] and which correlates with ecological momentary assessments of negative affect[13], was used to assess momentary change in hedonic capacity before and after L-DOPA and placebo. Patients were instructed to rate how much they agreed or disagreed with the 14 items phrased as "I would enjoy __" based on their ability to experience pleasure at the moment. Of the four possible response categories (Definitely Agree, Agree, Disagree, and Strongly Disagree), either of the Disagree responses received a score of 1 and either of the Agree responses received a score of 0. Thus, the SHAPS was scored as the sum of the 14 items so that total scores ranged from 0 to 14. A higher total SHAPS score indicated higher anhedonia. As the change in SHAPS score (post-pre) was not normal, values were natural log transformed for statistical analysis, with raw, untransformed means and values shown in displays. Motivation: The effort expenditure for reward task (EEfRT), a multi-trial game in which participants choose between two task difficulty levels on each trial in order to obtain monetary rewards[14] that is sensitive to pharmacological manipulation with dopaminergic drugs[15] and inflammation[16], was used to assess motivation. For all trials, participants made repeated manual button presses to raise the level of a virtual "bar" viewed onscreen. Participants were eligible to win the money allotted for each trial if they raised the bar to the "top" within the prescribed short period of time. Each trial presented the subject with a choice between two levels of task difficulty, a 'hard task' and an 'easy task'. Successful completion of hard-task trials required the subject to make 100 button presses using the non-dominant little finger within 21 seconds, while successful completion of easy-task trials required the subject to make 30 button presses using the dominant index finger within 7 seconds. Briefly, each trial presents the participant with a choice between an "easy" keypress task worth $1.00 and a "hard" keypress task worth a variable amount of reward ($1.24–$4.21). Participants are shown the amount the hard task is worth and the probability of winning (88%, 50%, and 12%) before making each choice. The primary outcome variable was choice of the hard task. Two "win" trials were randomly chosen for payout. Because subjects could only play for 20 minutes and the number of trials completed during that time varies from subject to subject, only the first 50 trials were used for consistency of analysis[14]. Analyzed subjects had less than 2% of trials that "timed-out" and at least 85% of trials were completed after L-DOPA administration. Choices were modeled using a generalized linear mixed-effects model (GLMM) with a logit link function for the binomial (hard/easy) outcome. Fixed effects were Probability (12%, 50%, or 88%), Amount ($1.24–$4.21), and their interactions (note that Probability and Amount interaction is referred to as the expected value of a reward). We also included fixed effects for Trial Number (0–50) and Session (1 or 2) to account for effects of fatigue and practice, consistent with prior analyses[17, 18].

Inflammatory markers. hsCRP: With our goal to not only examine dopaminergic mechanisms of inflammation impact on reward circuits, but also to establish fMRI and behavioral outcomes as well as screening methods to enroll MDD patients with high inflammation for future studies and clinical trials, mean plasma CRP concentrations measured during participant screening for eligibility for this and other studies in the Emory Behavioral Immunology Program (as described above) were used as the primary outcome variable to classify inflammation levels. For all patients, plasma hsCRP concentrations were analyzed by the Emory Medical Laboratory (EML), a fully licensed and CLIA-Certified clinical laboratory that provides diagnostic testing services for Emory Hospital and the Clinics, using SYNCHRON near infrared particle rate turbidimetry methods (Beckman Coulter) as described[19-21]. For 6 participants, at least one hsCRP value was analyzed at EML and at least one was analyzed using a hsCRP point of care (POC) test based on latex-enhanced immunoturbidimetric assay, using whole blood from a finger prick and previously validated in our lab for consistency with the clinical lab assay (hsCRP POC Test Kit, Diazyme Poway, CA)[22, 23]. For example, all values assessed by the POC method were above the assay detection limit (0.5 mg/L) and did not differ from values measured at EML for the same patients (5.1 +/- 2.8 for the POC versus 4.8 +/- 3.9 for EML, paired t=0.26, df=5, p=0.804), indicating a high level of both inter-lab and inter-subject consistency. Detection limits at EML varied and ranged from 0.03 to 1.0 mg/L; values under these limits were imputed as half of the detection value as in prior studies[24-26]. Of note, this had little impact on data analysis as all patients with one or more CRP value under the detection limit (n=12/40) had mean CRP ≤ 2mg/L. Inflammatory cytokines and their soluble receptors: To corroborate that mean screening CRP of >2 mg/L reflected that these patients also had higher concentrations of other inflammatory markers, inflammatory cytokines (IL-1beta, TNF and IL-6) and their soluble receptors (IL-1ra, TNFRII and IL-6sr) were assessed from batched EDTA plasma, which was collected in the morning after overnight fasting an average of 8 days before the first scan visit and stored at -80 degrees as previously described[19, 27, 28]. Briefly, multiplex magnetic bead-based immunoassays (R&D Systems, Minneapolis, MN) were used to quantify hs-cytokines and their receptors by Performance High Sensitivity Human and Screening Sensitivity Human MAP assays, respectively, on a Magpix Instrument (Luminex) with xPonent (Luminex) and Analyst (Millipore) software and a five-parameter curve fit. No variables were below the limits of assay detection (see **Table S2**), and coefficients of variation were reliably <10%. Cytokine and receptor values were inspected for outliers using the Grubbs test and data from one subject was excluded for extreme values in two markers. A composite score of inflammation was calculated as the sum of Z scores for concentrations of IL-1beta, TNF, IL-6 and their soluble receptors as previously described[19, 29]. Because the Z score centers the mean of each marker at 0 ±1 (mean, SD), classifying each participant as having "higher" (above 0) or "lower" (below 0) levels of each marker, when combined into an inflammatory composite score (as the sum of Z scores for each marker) it can be used as a continuous variable for comparison with other variables. See **Figure S2** for the contribution of Z scores for each individual marker to the relationship between the composite score and CRP > versus ≤ 2 mg/L (see also **Table 1**). Consistent with the use of CRP as a marker of both systemic and central inflammation, similarly recruited MDD patients with higher levels of CRP were found to have higher concentrations of these cytokines and receptors as assessed by the inflammatory composite score in both plasma and cerebrospinal fluid (CSF)[19], including in subjects with CRP > versus ≤ 2 mg/L (t=-4.1, df=79, p<0.001 in plasma; and t=-2.8, df=62, p>0.01 in CSF). Consistent with prior analyses, non-normal individual markers were natural log-transformed for subsequent statistical analyses using linear regression models with selection to identify which marker most significantly contributed to a relationship between the inflammatory composite score and the FC response to L-DOPA[30-32].

Plasma L-DOPA concentrations. Concentrations of L-DOPA and dopamine (to discriminate from L-DOPA) were determined by HPLC with tandem mass spectrometry detection using internal

standard as described[33-35]. Briefly, dialysate samples from 5-10 µl samples were injected into a LC system by automated injection (SIL-20ACHT, Shimadzu, Japan). Chromatographic separation was performed on a reversed phase analytical column, Synergi Max-RP (Phenomenex, Torrance, CA, USA). Detection was performed using a triple quadruple mass spectrometer and an API 4000 detector fitted with a Turbo Ion Spray interface (both AB Sciex, USA). Data were calibrated using calibration curve prepared in dialysate matrix and quantified using the Analyst data system (Applied Biosystems, version 1.4.2). As physiologic L-DOPA concentrations in plasma are very low (e.g., 10s of nM before versus 1000s of nM after L-DOPA administration in our preliminary data, see *Study Medication: Dosing* above)[6], L-DOPA concentrations were only measured after L-DOPA administration herein.

fMRI data acquisition and analyses. Imaging data were acquired at Emory's Center for Systems Imaging on a Siemens Prisma 3T scanner and 64-channel head coil. Anatomic images were obtained using a T1-weighted, magnetization prepared rapid gradient echo (MPRAGE) sequence[36] at 1 mm^3 resolution. Wakeful resting-state and task fMRI images were acquired by 2D gradient-echo EPI BOLD sequence with FOV = 210 × 210 mm, TR = 2000 ms, TE = 30 ms, resolution = 3.3 x 3.3 x 3.3 mm^3, flip angle = 90°, and #slices = 34. Resting fMRI was acquired both before and after L-DOPA or placebo administration using phase-encoding directions of opposite polarity (anterior-posterior) for distortion correction over ~10 minutes[37]. Data were analyzed with standard preprocessing protocols in AFNI (http://afni.nimh.nih.gov/), including slice-timing correction, realignment/motion-correction, anat-to-epi co-registration, and 5 mm spatial smoothing. Resting BOLD time series were additionally processed to minimize artifacts from head motion, respiration, cardiac pulsation, and hardware using the ANATICOR method[38] by performing motion and outlier censoring (aka scrubbing), nuisance regression (motion parameters, as well as averaged signals from cerebrospinal fluid and local white matter), and band pass filtering ($0.009\text{Hz} < f < 0.08\text{Hz}$). For the signal censoring of resting-state fMRI, timepoints with temporal outliers (~5.5 times median absolute deviation) or excessive head motion (frame displacement > 0.3 mm) were excluded[27, 39]. A frame displacement threshold of > 0.9 mm was used for task-based fMRI as this analysis assumed a specific BOLD response profile (hence less sensitive to noise) and was shorter on effective temporal length (see below for beta series derivation). After preprocessing, individuals' 4D fMRI data were spatially normalized into the standard stereotaxic space Montreal Neurological Institute (MNI) template, with 2 mm isotropic resolution. Participants were also screened from subsequent group-level statistics based on parameters generated in the preprocess pipeline[39]. For resting fMRI, subsequently "analyzable" subjects satisfied a logical conjunction of 3 conditions: (i) >5 minutes of signal length after motion and outlier censoring, (ii) <2 mm of max pair-wise displacement on Euclidean norm of the 6-dimensional head motion estimates, and (iii) <15% of censored/excluded time points in the scan. For task fMRI, due to assumed response profiles and less effective temporal samples, the conjunction was loosened by dropping condition (i) and using 3 mm (EPI voxel size) in condition (ii).

For both resting-state and task-based (see MID below) fMRI analyses, VS-vmPFC FC was assessed using a targeted, *a priori* seed-to-ROI approach. Subject-level Fisher's normalized Z-scores were extracted for FC between a 3 mm radius spherical seed centered on the left VS (including nucleus accumbens; MNI coordinates: x = −9, y = 9, z = −8)[40, 41] and a ROI in the vmPFC (MNI coordinates: x = 0, y = 44, z = −8 and volume = 1408 mm^3, encompassing parts of BA11 and ventral BA32 of the anterior cingulate cortex) previously associated with neural activation to receipt of reward in neuroimaging meta-analysis[42] and used in our previous work[27-29, 43]. This unbiased approach was chosen as the primary analytic strategy for this study to ensure rigor, reproducibility, and potential application of results to future trials because the seeds and ROI were predefined as opposed to derived from whole-brain and data-driven analyses that may be specific to the current sample. Primary hypotheses and thus analyses also focused on left VS

because studies examining effects of inflammation on striatal neural activation, neurotransmitters and metabolism, as well as numerous of our own studies using similar VS-vmPFC FC analyses[27, 29, 44], have reported greater effects in or findings lateralized to the left side[45-47]. Exploratory results for right VS for the primary analysis of resting-state FC are presented below.

Monetary Incentive Delay (MID) Task. The MID was used to assess VS-vmPFC FC during reward anticipation[48-50]. Although this task has established test-retest reliability[1], MID was only conducted after L-DOPA or placebo administration to avoid potential same-day carry over effects. Monetary outcome depended on patient performance in a simple reaction time task by pressing a button in response to a visual target stimulus. The "anticipatory delay," ~4000 ms, which occurs after presentation of a pseudo-randomly distributed cue to inform participants whether a given trial will allow them to win or lose money (reward: +$; loss: -$; no incentive: 0$; averaging ~$2) but prior to the target stimulus, has reliably been shown to robustly activate ventral striatum[51-53]. Participants completed 2 functional runs of 70 trials each (140 trials total) over ~20 minutes[48, 50]. Task-based FC during each anticipation condition was assessed using beta-series correlation[54], a powerful and sensitive method to estimate task-modulated FC by assessing functional networks during distinct stages of a task[55, 56] which has been used before with MID[48, 50]. A GLM design matrix using separate regressors was constructed to model hemodynamic responses during anticipatory conditions (gain, neutral, or loss cues) separately for each single trial. This iterative procedure was repeated until the entire cue-specific beta-series was generated. These voxel-wise beta-series were treated as time courses in subsequent seed-based connectivity analysis (cross-correlation) for deriving Fisher's Z scores (see above). The relative difference in Z scores between cue conditions indicated the estimated magnitude of context-modulated VS-vmPFC FC. The contrast of interest was FC during reward anticipation following gain > neutral cues.

MRI procedures: adverse events and data quality. As described above, three patients withdrew from the study after experiencing symptoms of anxiety or claustrophobia during the first scan. Two additional subjects who were able to ultimately complete the study reported anxiety that required temporary termination of scans. In addition to this high rate of discomfort and anxiety there was a significant proportion of subjects with excessive motion artifact in at least one post-L-DOPA or placebo challenge scan (15/56, ~27%). This was despite instructions and frequent reminders to participants to remain still and monitoring of visible motion by study staff and MRI technicians. Although the patients with analyzable data for this report were highly representative of those who completed the parent study (see **Table S1**), it should be noted, and consistent with prior MDD studies[27, 57], the patient sample was comprised primarily of women (~70%) with higher BMI (mean 29.6 $\pm$6). Fit tests were conducted for patients with BMI >35, and while all subjects studied endorsed physical comfort in the scanner, there was some concern for possible movement artifact from normal breathing in larger patients. Use of multi-echo acquisition protocols in future studies may reduce motion artifact in similar patient samples in future studies.

Power Calculations. Based on anticipated effect sizes for the VS-vmPFC FC response to L-DOPA (ES; Cohen's d) >1.0, the parent study planned to enroll 60 patients with a range of CRP concentrations with >90% power to detect higher FC following L-DOPA with respect to placebo in patients with higher levels of inflammation at α=0.05 when controlling for covariates. For the analysis of post-L-DOPA FC when controlling for placebo and covariates in 40 patients (n=19 with CRP >2 mg/L), the observed ES=1.01 provided a post-hoc power >87% for analysis of the primary outcome.

SUPPLEMENTAL RESULTS

Adverse events: Of the 56 patients that completed study visits with both L-DOPA and placebo challenge, 22 (39%) experienced at least one AE either before administration of study medication (16%) or after placebo (23%), and 34 (61%) experienced at least one AE after L-DOPA. All AEs reported during study visits either before study medication, after placebo or after L-DOPA were mild to moderate, resolved before the end of the study visit, and were similar across conditions: 1) anxiety, headache, dizziness, drowsiness, and nausea were reported before administration of study medication; 2) anxiety, headache, dizziness, vomiting, muscle spasms, muscle stiffness, numbness/paresthesia, elevated blood pressure, lower back pain, double vision, feeling "floaty," and diarrhea were reported after placebo; and 3) anxiety, headache, dizziness, drowsiness, nausea, vomiting, muscle stiffness, numbness/paresthesia, elevated blood pressure, sensation of increased heart rate/heart fluttering, hypotension, lightheadedness, vertigo/spinning, feeling hot/flushed, shortness of breath, disorientation, depersonalization, feeling faint, dry eyes, dry cough, and nasal pruritis were reported after L-DOPA. The most common AE reported after L-DOPA was nausea, which occurred in 46% of patients. Compared to the overall study completers (n=56), patients with analyzable scans after L-DOPA and placebo (n=40) report AEs at similar rates after L-DOPA (62.5 versus 61%) and before the administration of study medication (15 versus 16%) but did report a lower rate of AEs after placebo (12.5 versus 23%). Of the analyzed sample, patients with CRP > (n=19) versus ≤ (n=21) 2 mg/L reported more AEs after placebo (21 versus 5% of patients) and less AEs after L-DOPA (53 versus 71% of patients), but these rates did not significantly differ (Fisher's Exact test [1,39]=2.4, p=0.172 and χ^2[1,39]=1.5, p=0.220, respectively).

Classification of patients as CRP > versus ≤ 2 mg/L based on the VS-vmPFC FC response to L-DOPA: As patients with CRP > 2 mg/L had positive and significantly higher VS-vmPFC FC after L-DOPA challenge with respect to placebo in linear models (**Figure 2**), logistic regression and receiver operating characteristic (ROC) analyses were also used to determine whether the FC response to L-DOPA (FC Z scores, post minus pre) was able to classify patients as having higher CRP based on > 2 mg/L versus other cut-points (1 or 3 mg/L). Indeed, the VS-vmPFC FC response to L-DOPA significantly predicted whether or not patients had CRP > 2 mg/L (OR=62.0; 95%CI=1.3,2999; p=0.037; and p=0.028 when controlling for placebo response and covariates), but not > CRP 1 or 3 (p=0.543 and 0.098). Similarly, in ROC analysis, the VS-vmPFC FC response to L-DOPA significantly classified patients as having CRP > 2 mg/L (but not other CRP cut-points) with sensitivity 86% and specificity 59% (p=0.013; **Figure S3**). In this analysis, the sensitive point for FC response to classify CRP levels was ~0 (FC response=-0.005), indicating that a positive response to L-DOPA (change in FC >0) classified patients as having CRP >2 mg/L.

Exploratory results for right VS to vmPFC resting-state FC: Consistent with our hypothesis that inflammation by L-DOPA effects would be observed primarily for the left VS to vmPFC FC, only a trend for an interaction effect of CRP (either as a continuous variable or at the cut-point of CRP > 2 mg/L) was observed for right VS-vmPFC FC response to L-DOPA (post minus pre, n=31) versus placebo with or without covariates (all p>0.195). In GEE analyses, there was also no difference in post-L-DOPA FC (n=40) patients with CRP > versus ≤ 2 mg/L when controlling for placebo either with (or without covariates, B=0.02 to 0.05, p=0.435 to 0.778).

Behavior after L-DOPA with respect to placebo in patients with CRP > versus ≤ 2 mg/L: Mean ± S.D. for behavioral measures by treatment condition and CRP group are shown in **Table 1**. While there were trends for motivation (percent hard choices in the 50% reward probability condition) to be lower and anhedonia (SHAPS scores) to be higher in patients with CRP > versus ≤ 2 mg/L after placebo (p=0.091 to 0.202; see **Table 1**), there was no effect of L-DOPA

versus placebo challenge or an interaction with CRP observed in linear models including within subject (treatment) and between subject (CRP either continuous concentrations or level, > versus ≤ 2 mg/L) variables for either outcome to warrant further comparison (all p>0.152). There were also no differential effects on motivation or anhedonia response after challenge with L-DOPA with respect to placebo based on CRP level. For example, in GEE analyses there were no differences in the proportion of hard choices in either the 50 or 88% reward probability conditions in patients with CRP > versus ≤ 2 mg/L after L-DOPA when controlling for placebo either with (B=-0.02 to -0.31, p=0.512 to 0.855) or without covariates (B=-0.01 to -0.02, p=0.801 to 0.855). Similarly, there was no difference in the change in SHAPS (post minus pre total scores) to L-DOPA with respect to placebo, either with or without covariates (B=-0.14 to -0.25, p=0.439 to 0.680). However, there were relationships between these behaviors and the primary outcome of VS-vmPFC rsFC (see **Table 2** and **Figure 3** in manuscript). Of note, change in SHAPS did not correlate with post-L-DOPA or placebo performance on EEfRT. There was however a significant treatment by CRP interaction for the relationship between post-treatment anhedonia (SHAPS scores) and proportion of hard choices at the 88% (but not at 50 or 12%) reward probability conditions (r=-0.29, df=71, p=0.013) whereby a trend for post-L-DOPA (but not placebo) anhedonia to inversely correlate with motivation was seen in patients with CRP >2 mg/L (r=-0.38, p=0.135), while a significant positive correlation was found in patients with CRP ≤2 mg/L (r=0.60, p=0.004).

Control of study-related variables including variability of L-DOPA concentrations, fMRI distortion correction, presence of AEs, and treatment order: As plasma concentrations of L-DOPA exhibited both inter-subject variability and differences based on the carbidopa dose (see above), L-DOPA concentrations were controlled for in all significant findings, along with other study-related variables that may have influenced results. These included AEs (coded 0 for no AEs, 1 for AEs after placebo only, 2 for AEs after L-DOPA only, and 3 for AEs after both placebo and L-DOPA), treatment order (coded 0 or 1 for receipt of either placebo or L-DOPA at the first visit), and a variable related to fMRI distortion correction[37] as scans with phase-encoding directions of opposite polarity were not available for either the post-L-DOPA or post-placebo scans in 5 of 40 participants (coded 1 versus 0 for all other participants). These variables were included in significant models where applicable separately from clinical covariates to avoid overfitting.

All significant findings reported in the manuscript remained p<0.05 when controlling for these variables including: interaction of treatment with CRP (as a continuous variable) on rsFC response to L-DOPA and placebo in GLM (F[1,25]=4.4, p=0.046), increased VS-vmPFC rsFC after L-DOPA with respect to placebo in patients with CRP > versus ≤ 2 mg/L for both the L-DOPA response (post minus pre, n=31; B=0.22, SE(B)=0.06, p<0.001) and for post-L-DOPA rsFC in all 40 patients (B=0.13, SE(B)=0.04, p=0.004), and VS-vmPFC tbFC during reward anticipation in the MID (gain versus neutral cues; B=0.13, SE(B)=0.05, p=0.009). The interaction of treatment with CRP level on the relationship between post-challenge rsFC and anhedonia response (SHAPS scores, post minus pre) also remained significant (r=-0.23, df=70, p=0.044) when controlling for these variables.

S Figures and Tables

Table S1. Demographic and clinical characteristics of the patients that were studied (n=56) and those with analyzable rsFC data (n=40).

		Studied (N=56)	Analyzed (N=40)
**Age**	Mean (SD)	37.6 (11.0)	36.4 (11.3)
**Sex**	N (%) Male	15 (26.8%)	12 (30.0%)
**Race**	N (%) White	31 (55.4%)	24 (60.0%)
**BMI**	Mean (SD)	29.6 (6.0)	28.6 (5.7)
**CRP mg/L**	Median (IQR)	2.1 (2.6)	2.0 (2.6)
**CRP > 2 mg/L**	N (%)	28 (50.0%)	19 (47.5%)
**HAM-D**	Mean (SD)	22.2 (3.9)	21.8 (3.5)
**Plasma L-DOPA (nM)**	Median (IQR)	6600 (5470)	6005 (4827.5)
**Plasma DA (nM)**	Median (IQR)	9.5 (5.4)	9.3 (5.4)

Abbreviations: BMI, body mass index; CRP, C-reactive protein; DA, dopamine; FC, functional connectivity; HAM-D, Hamilton Depression Rating Scale; IQR, interquartile range; L-DOPA, levodopa; SD, standard deviation.

Table S2. Detection limits for MAP assays for plasma cytokines and their soluble receptors.

Variable	MDL (pg/ml)
IL-1beta	0.2
TNF	0.4
IL-6	0.2
IL-1ra	14.7
sTNFRII	5.5
IL-6sr	53.2

IL, interleukin; MAP, multi-analyte profiling; MDL, method detection limit; ra, receptor antagonist; sr, soluble receptor; sTNFRII, soluble tumor necrosis factor receptor type II; TNF, tumor necrosis factor.

Figure S1. Flow chart of patient screening, enrollment, and analysis.
Abbreviations: AE - adverse event; EEfRT - effort expenditure task; fMRI - functional magnetic resonance imaging; L-DOPA - levodopa; MID - monetary incentive delay.

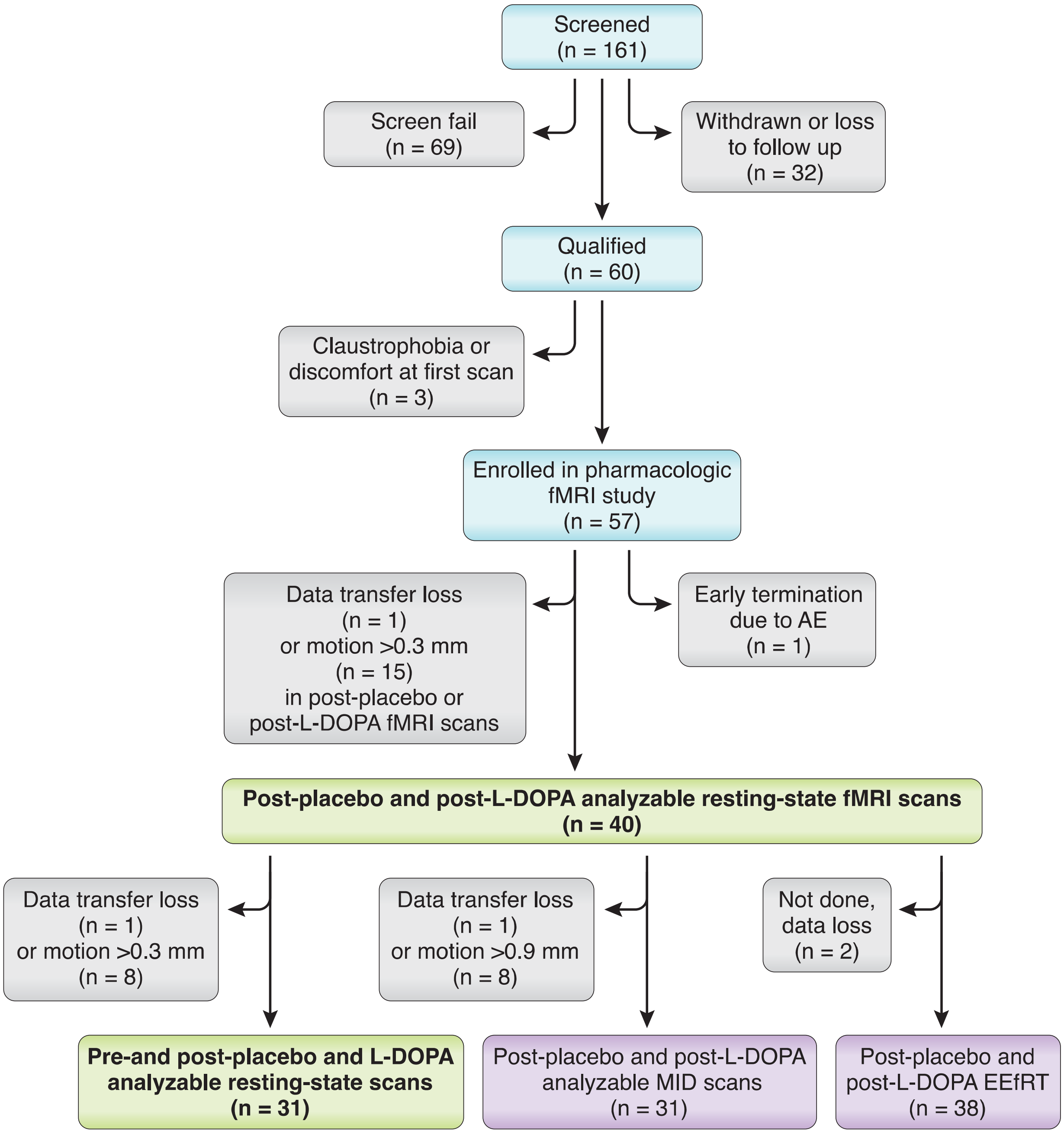

Figure S2. Inflammatory composite score and the contributing Z scores for inflammatory cytokines and their soluble receptors in plasma of MDD patients with CRP > versus ≤ 2 mg/L. Data are presented as mean ± standard error. *p <0.05. Abbreviations: a - antagonist; CRP - C-reactive protein; IL - interleukin; r - receptor; s - soluble; TNF - tumor necrosis factor

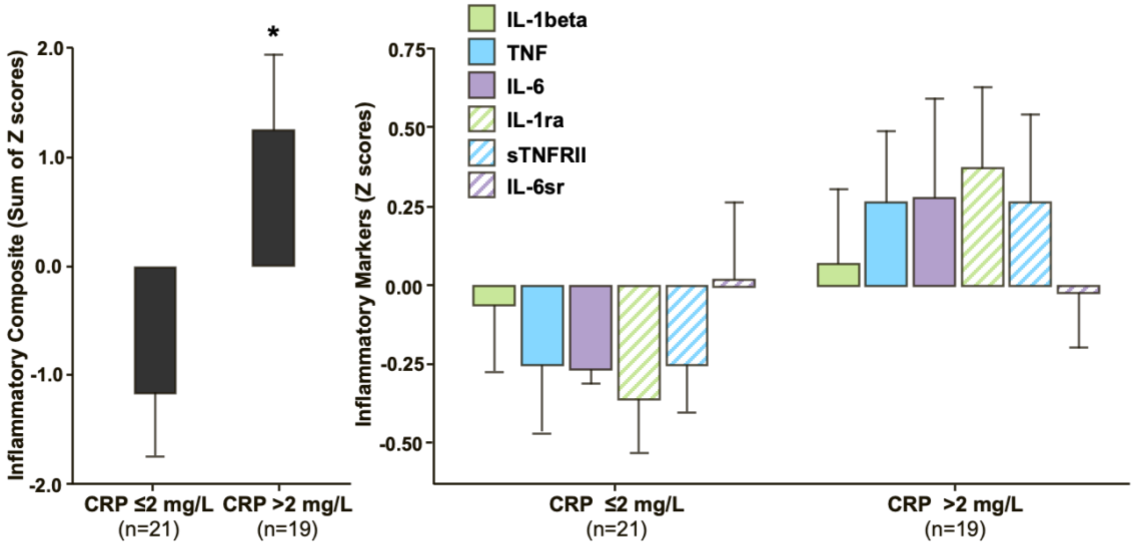

Figure S3. Receiver operating characteristic (ROC) curve for the significant classification of CRP >2 mg/L (but not other cut-points) by the VS-vmPFC rsFC response to L-DOPA challenge. Abbreviations: CRP - C-reactive protein; FC - functional connectivity; rs - resting state.

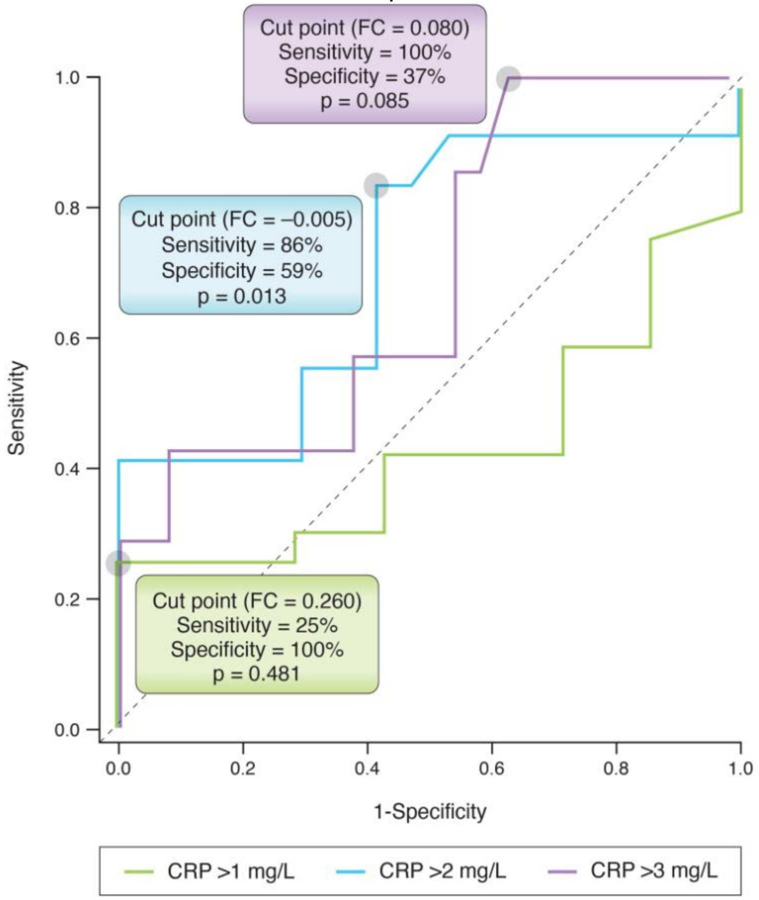

S References

1. Wu CC, Samanez-Larkin GR, Katovich K, Knutson B. Affective traits link to reliable neural markers of incentive anticipation. *Neuroimage* 2014; **84:** 279-289.

2. Wardle MC, Treadway MT, Mayo LM, Zald DH, de Wit H. Amping Up Effort: Effects of d-Amphetamine on Human Effort-Based Decision-Making. *Journal of Neuroscience* 2011; **31**(46)**:** 16597-16602.

3. Soder HE, Cooper JA, Lopez-Gamundi P, Hoots JK, Nunez C, Lawlor VM *et al.* Dose-response effects of d-amphetamine on effort-based decision-making and reinforcement learning. *Neuropsychopharmacology : official publication of the American College of Neuropsychopharmacology* 2021; **46**(6)**:** 1078-1085.

4. Kelly C, de Zubicaray G, Di Martino A, Copland DA, Reiss PT, Klein DF *et al.* L-dopa modulates functional connectivity in striatal cognitive and motor networks: a double-blind placebo-controlled study. *The Journal of neuroscience : the official journal of the Society for Neuroscience* 2009; **29**(22)**:** 7364-7378.

5. Delaveau P, Salgado-Pineda P, Wicker B, Micallef-Roll J, Blin O. Effect of levodopa on healthy volunteers' facial emotion perception: an FMRI study. *Clinical neuropharmacology* 2005; **28**(6)**:** 255-261.

6. Buhmann C, Glauche V, Sturenburg HJ, Oechsner M, Weiller C, Buchel C. Pharmacologically modulated fMRI--cortical responsiveness to levodopa in drug-naive hemiparkinsonian patients. *Brain* 2003; **126**(Pt 2)**:** 451-461.

7. Shiner T, Symmonds M, Guitart-Masip M, Fleming SM, Friston KJ, Dolan RJ. Dopamine, Salience, and Response Set Shifting in Prefrontal Cortex. *Cerebral cortex* 2015; **25**(10)**:** 3629-3639.

8. Okereke CS. Role of integrative pharmacokinetic and pharmacodynamic optimization strategy in the management of Parkinson''s disease patients experiencing motor fluctuations with levodopa. *Journal of pharmacy & pharmaceutical sciences : a publication of the Canadian Society for Pharmaceutical Sciences, Societe canadienne des sciences pharmaceutiques* 2002; **5**(2)**:** 146-161.

9. Djaldetti R, Giladi N, Hassin-Baer S, Shabtai H, Melamed E. Pharmacokinetics of etilevodopa compared to levodopa in patients with Parkinson's disease: an open-label, randomized, crossover study. *Clinical neuropharmacology* 2003; **26**(6)**:** 322-326.

10. Felger JC, Li Z, Haroon E, Woolwine BJ, Jung MY, Hu X *et al.* Inflammation is associated with decreased functional connectivity within corticostriatal reward circuitry in depression. *Mol Psychiatry* 2015.

11. Alexander GM, Schwartzman RJ, Grothusen JR, Gordon SW. Effect of plasma levels of large neutral amino acids and degree of parkinsonism on the blood-to-brain transport of levodopa in naive and MPTP parkinsonian monkeys. *Neurology* 1994; **44**(8)**:** 1491-1499.

12. Nakonezny PA, Carmody TJ, Morris DW, Kurian BT, Trivedi MH. Psychometric evaluation of the Snaith-Hamilton pleasure scale in adult outpatients with major depressive disorder. *Int Clin Psychopharmacol* 2010; **25**(6)**:** 328-333.

13. Murray L, Israel ES, Balkind EG, Pastro B, Lovell-Smith N, Lukas SE *et al.* Multi-modal assessment of reward functioning in adolescent anhedonia. *Psychol Med* 2022**;** Jun 17: 1-10.

14. Treadway MT, Buckholtz JW, Schwartzman AN, Lambert WE, Zald DH. Worth the 'EEfRT'? The effort expenditure for rewards task as an objective measure of motivation and anhedonia. *PLoS One* 2009; **4**(8)**:** e6598.

15. Treadway MT, Buckholtz JW, Cowan RL, Woodward ND, Li R, Ansari MS *et al.* Dopaminergic mechanisms of individual differences in human effort-based decision-making. *J Neurosci* 2012; **32**(18)**:** 6170-6176.

16. Boyle CC, Kuhlman KR, Dooley LN, Haydon MD, Robles TF, Ang YS *et al.* Inflammation and dimensions of reward processing following exposure to the influenza vaccine. *Psychoneuroendocrinology* 2019; **102:** 16-23.

17. Wardle MC, Treadway MT, de Wit H. Caffeine increases psychomotor performance on the effort expenditure for rewards task. *Pharmacol Biochem Behav* 2012; **102**(4)**:** 526-531.

18. Wardle MC, Treadway MT, Mayo LM, Zald DH, de Wit H. Amping Up Effort: Effects of d-Amphetamine on Human Effort-Based Decision-Making. *Journal of Neuroscience* 2011; **31**(46)**:** 16597-16602.

19. Felger JC, Haroon E, Patel TA, Goldsmith DR, Wommack EC, Woolwine BJ *et al.* What does plasma CRP tell us about peripheral and central inflammation in depression? *Mol Psychiatry* 2020; **25**(6)**:** 1301-1311.

20. Lin E, Phillips LS, Ziegler TR, Schmotzer B, Wu K, Gu LH *et al.* Increases in adiponectin predict improved liver, but not peripheral, insulin sensitivity in severely obese women during weight loss. *Diabetes* 2007; **56**(3)**:** 735-742.

21. Su S, Lampert R, Zhao J, Bremner JD, Miller A, Snieder H *et al.* Pleiotropy of C-reactive protein gene polymorphisms with C-reactive protein levels and heart rate variability in healthy male twins. *Am J Cardiol* 2009; **104**(12)**:** 1748-1754.

22. Zheng H, Bergamino M, Ford BN, Kuplicki R, Yeh FC, Bodurka J *et al.* Replicable association between human cytomegalovirus infection and reduced white matter fractional anisotropy in major depressive disorder. *Neuropsychopharmacology : official publication of the American College of Neuropsychopharmacology* 2021; **46**(5)**:** 928-938.

23. Slysz J, Slysz G, Slysz A, Burr J. The Accuracy and Precision of the Diazyme SMART 700-300 Analyzer for HbA1c and hs-CRP. *The Health & Fitness Journal of Canada* 2017; **10**(2)**:** 77-83.

24. Dutcher JM, Boyle CC, Eisenberger NI, Cole SW, Bower JE. Neural responses to threat and reward and changes in inflammation following a mindfulness intervention. *Psychoneuroendocrinology* 2021; **125:** 105114.

25. Ancelin ML, Farré A, Carrière I, Ritchie K, Chaudieu I, Ryan J. C-reactive protein gene variants: independent association with late-life depression and circulating protein levels. *Transl Psychiatry* 2015; **5**(1)**:** e499.

26. Ferrie JE, Kivimäki M, Akbaraly TN, Singh-Manoux A, Miller MA, Gimeno D *et al.* Associations between change in sleep duration and inflammation: findings on C-reactive protein and interleukin 6 in the Whitehall II Study. *Am J Epidemiol* 2013; **178**(6)**:** 956-961.

27. Felger JC, Li Z, Haroon E, Woolwine BJ, Jung MY, Hu X *et al.* Inflammation is associated with decreased functional connectivity within corticostriatal reward circuitry in depression. *Mol Psychiatry* 2016; **21**(10)**:** 1358-1365.

28. Yin L, Xu X, Chen G, Mehta ND, Haroon E, Miller AH *et al.* Inflammation and decreased functional connectivity in a widely-distributed network in depression: Centralized effects in the ventral medial prefrontal cortex. *Brain Behav Immun* 2019; **80:** 657-666.

29. Mehta ND, Stevens JS, Li Z, Gillespie CF, Fani N, Michopoulos V *et al.* Inflammation, reward circuitry and symptoms of anhedonia and PTSD in trauma-exposed women. *Soc Cogn Affect Neurosci* 2020; **15**(10)**:** 1046-1055.

30. Haroon E, Felger JC, Woolwine BJ, Chen X, Parekh S, Spivey JR *et al.* Age-related increases in basal ganglia glutamate are associated with TNF, reduced motivation and decreased psychomotor speed during IFN-alpha treatment: Preliminary findings. *Brain Behav Immun* 2015; **46:** 17-22.

31. Raison CL, Borisov AS, Majer M, Drake DF, Pagnoni G, Woolwine BJ *et al.* Activation of central nervous system inflammatory pathways by interferon-alpha: relationship to monoamines and depression. *Biol Psychiatry* 2009; **65**(4)**:** 296-303.

32. Torres MA, Pace TW, Liu T, Felger JC, Mister D, Doho GH *et al.* Predictors of depression in breast cancer patients treated with radiation: role of prior chemotherapy and nuclear factor kappa B. *Cancer* 2013; **119**(11)**:** 1951-1959.

33. Lindemann L, Meyer CA, Jeanneau K, Bradaia A, Ozmen L, Bluethmann H *et al.* Trace amine-associated receptor 1 modulates dopaminergic activity. *J Pharmacol Exp Ther* 2008; **324**(3)**:** 948-956.

34. Pehrson AL, Cremers T, Betry C, van der Hart MG, Jorgensen L, Madsen M *et al.* Lu AA21004, a novel multimodal antidepressant, produces regionally selective increases of multiple neurotransmitters--a rat microdialysis and electrophysiology study. *Eur Neuropsychopharmacol* 2013; **23**(2)**:** 133-145.

35. Felger JC, Mun J, Kimmel HL, Nye JA, Drake DF, Hernandez CR *et al.* Chronic interferon-alpha decreases dopamine 2 receptor binding and striatal dopamine release in association with anhedonia-like behavior in nonhuman primates. *Neuropsychopharmacology* 2013; **38**(11)**:** 2179-2187.

36. Mugler JP, 3rd, Brookeman JR. Three-dimensional magnetization-prepared rapid gradient-echo imaging (3D MP RAGE). *Magn Reson Med* 1990; **15**(1)**:** 152-157.

37. Birn RM, Molloy EK, Patriat R, Parker T, Meier TB, Kirk GR *et al.* The effect of scan length on the reliability of resting-state fMRI connectivity estimates. *Neuroimage* 2013; **83:** 550-558.

38. Jo HJ, Saad ZS, Simmons WK, Milbury LA, Cox RW. Mapping sources of correlation in resting state FMRI, with artifact detection and removal. *NeuroImage* 2010; **52**(2)**:** 571-582.

39. Power JD, Mitra A, Laumann TO, Snyder AZ, Schlaggar BL, Petersen SE. Methods to detect, characterize, and remove motion artifact in resting state fMRI. *NeuroImage* 2014; **84:** 320-341.

40. Di Martino A, Scheres A, Margulies DS, Kelly AM, Uddin LQ, Shehzad Z *et al.* Functional connectivity of human striatum: a resting state FMRI study. *Cerebral cortex (New York, NY : 1991)* 2008; **18**(12)**:** 2735-2747.

41. Furman DJ, Hamilton JP, Gotlib IH. Frontostriatal functional connectivity in major depressive disorder. *Biology of mood & anxiety disorders* 2011; **1**(1)**:** 11.

42. Diekhof EK, Kaps L, Falkai P, Gruber O. The role of the human ventral striatum and the medial orbitofrontal cortex in the representation of reward magnitude - an activation likelihood estimation meta-analysis of neuroimaging studies of passive reward expectancy and outcome processing. *Neuropsychologia* 2012; **50**(7)**:** 1252-1266.

43. Mehta ND, Haroon E, Xu X, Woolwine BJ, Li Z, Felger JC. Inflammation negatively correlates with amygdala-ventromedial prefrontal functional connectivity in association with anxiety in patients with depression: Preliminary results. *Brain Behav Immun* 2018; **73:** 725-730.

44. Goldsmith DR, Bekhbat M, Le NA, Chen X, Woolwine BJ, Li Z *et al.* Protein and gene markers of metabolic dysfunction and inflammation together associate with functional connectivity in reward and motor circuits in depression. *Brain Behav Immun* 2020; **88:** 193-202.

45. Capuron L, Pagnoni G, Demetrashvili MF, Lawson DH, Fornwalt FB, Woolwine B *et al.* Basal ganglia hypermetabolism and symptoms of fatigue during interferon-alpha therapy. *Neuropsychopharmacology* 2007; **32**(11)**:** 2384-2392.

46. Haroon E, Woolwine BJ, Chen X, Pace TW, Parekh S, Spivey JR *et al.* IFN-alpha-induced cortical and subcortical glutamate changes assessed by magnetic resonance spectroscopy. *Neuropsychopharmacology* 2014; **39**(7)**:** 1777-1785.

47. Eisenberger NI, Berkman ET, Inagaki TK, Rameson LT, Mashal NM, Irwin MR. Inflammation-induced anhedonia: endotoxin reduces ventral striatum responses to reward. *Biol Psychiatry* 2010; **68**(8)**:** 748-754.

48. Jung WH, Kang DH, Kim E, Shin KS, Jang JH, Kwon JS. Abnormal corticostriatal-limbic functional connectivity in obsessive-compulsive disorder during reward processing and resting-state. *Neuroimage Clin* 2013; **3:** 27-38.

49. Admon R, Nickerson LD, Dillon DG, Holmes AJ, Bogdan R, Kumar P *et al.* Dissociable cortico-striatal connectivity abnormalities in major depression in response to monetary gains and penalties. *Psychol Med* 2015; **45**(1)**:** 121-131.

50. Ye Z, Hammer A, Camara E, Munte TF. Pramipexole modulates the neural network of reward anticipation. *Human brain mapping* 2011; **32**(5)**:** 800-811.

51. Knutson B, Fong GW, Adams CM, Varner JL, Hommer D. Dissociation of reward anticipation and outcome with event-related fMRI. *Neuroreport* 2001; **12**(17)**:** 3683-3687.

52. Treadway MT, Buckholtz JW, Zald DH. Perceived stress predicts altered reward and loss feedback processing in medial prefrontal cortex. *Front Hum Neurosci* 2013; **7:** 180.

53. Knutson B, Bhanji JP, Cooney RE, Atlas LY, Gotlib IH. Neural responses to monetary incentives in major depression. *Biol Psychiatry* 2008; **63**(7)**:** 686-692.

54. Rissman J, Gazzaley A, D'Esposito M. Measuring functional connectivity during distinct stages of a cognitive task. *Neuroimage* 2004; **23**(2)**:** 752-763.

55. Fornito A, Yoon J, Zalesky A, Bullmore ET, Carter CS. General and specific functional connectivity disturbances in first-episode schizophrenia during cognitive control performance. *Biol Psychiatry* 2011; **70**(1)**:** 64-72.

56. Cisler JM, Bush K, Steele JS. A comparison of statistical methods for detecting context-modulated functional connectivity in fMRI. *Neuroimage* 2014; **84:** 1042-1052.

57. Raison CL, Rutherford RE, Woolwine BJ, Shuo C, Schettler P, Drake DF *et al.* A randomized controlled trial of the tumor necrosis factor antagonist infliximab for treatment-resistant depression: the role of baseline inflammatory biomarkers. *JAMA Psychiatry* 2013; **70**(1)**:** 31-41.
